# Repair of injured right inferior pulmonary vein during mitral valve replacement

**DOI:** 10.1186/1749-8090-4-64

**Published:** 2009-11-07

**Authors:** Efstratios Apostolakis, Vassilios N Leivaditis, Antonios Kallikourdis, Panagiotis Dedeilias

**Affiliations:** 1 Cardiothoracic Surgery Department, Patras University Medical School, Patras, Greece; 2 Aberdeen Royal Infirmary, Aberdeen, UK; 3 1st Cardiac Surgery Department, Evangelismos General Hospital, Athens, Greece

## Abstract

During mitral valve surgery right pulmonary veins injury, subsequent to excessive traction (for better exposure of the mitral apparatus), is often unavoidable. This is more likely in patients with small left atrium. This common complication may cause severe intraoperative bleeding, while its surgical repair may lead to complications such as late stenosis or obstruction of the pulmonary veins. This injury should be early detected, before left atriotomy closing, and it is suggested to be repaired using a patch so as to avoid any possible late constriction.

We describe a case -to our knowledge, the first reported in the literature- of intraoperatively injured right inferior pulmonary vein in a patient who underwent mitral valve replacement. As outlined we propose that the ostium of the right inferior pulmonary vein can be repaired by using autologous pericardial patch, incorporated in the completion of left atriotomy closure.

## Introduction

The exposure of mitral valve during its repair or replacement is usually obtained through the Sondergaard's groove method. Typically the atriotomy comprises partial detachment and retraction of the right atrium from the left followed by incision at the midpoint between the right inferior pulmonary vein's (RIPV) take off and the interatrial groove [1]. During atrial traction manipulations, the risk of an inadvertent injury to the posterior wall of the left atrium or to the right pulmonary veins is substantial. In the cases of a small left atrium or short pulmonary veins the intraoperative risk of such injury is substantial. The effort to controll the bleeding may usually result in further injury of pulmonary veins, in lung parenchyma injury or in stenosis of corresponding pulmonary vein's outlet. We report a case of a successfully repaired injury of the RIPV during mitral valve replacement.

## A case report

**Figure 1:**
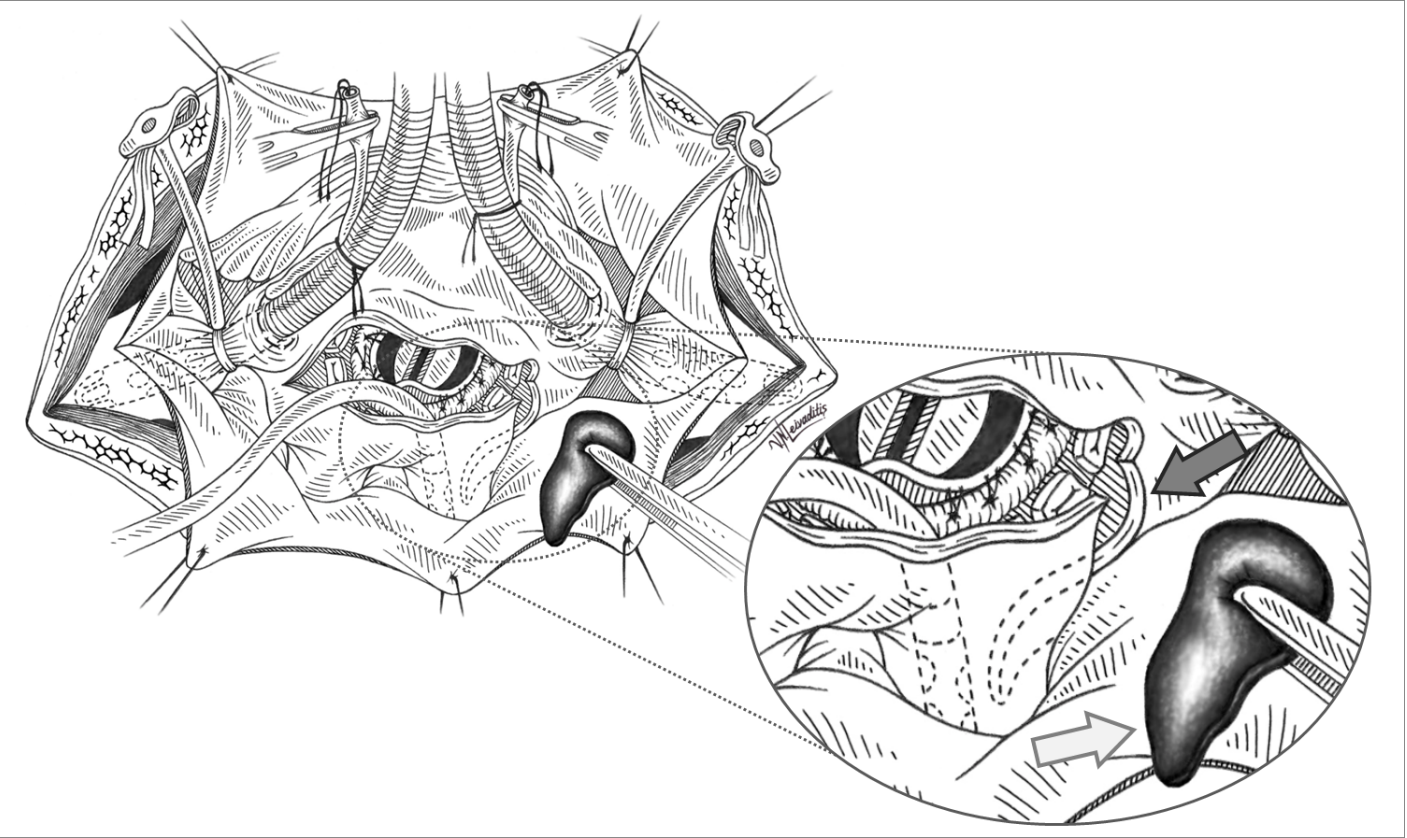
**Left Atrium rupture is extended to the lateral wall of the right inferior pulmonary vein (grey arrow)**. Suture effort is hard without inserting the vent in the pulmonary vein due to overflooting of blood in the surgical field. Repair will be performed using autologus pericardial patch (white arrow) to avoid possible stenosis of the vein.

A 77-year old diabetic male patient, ex smoker with COPD, had a 4 month history of severe mitral regurgitation (4+/4+) with a subsequent couple of episodes of pulmonary oedema. He referred to our University Hospital Department from the cardiology ward for urgent surgery. The preoperative echo revealed severe degenerative mitral valve disease, ruptured mainly to the posterior leaflet's cordae (P_2 _- P_3_) as well as the anterior leaflet's cordae (A_1_). The left atrium was found to be 42 mm in diameter. The patient was operated using typical bicaval cannulation for the Cardiopulmonary Bypass (CPB) circuit and systematic cooling to 30°C. The left atrium incision was made just inferior to the interatrial groove and in front of the right upper pulmonary vein's outlet (Sondergaard's incision). The Mitral Valve exposure was obtained using a Fraser - type retractor. Ruptures of the primary tendon cordae in P_2 _and partially P_3 _segments as well as in A1 were identified. The valve leaflets were not considered repairable due to the extension of the cordae ruptures and the degeneration of the leaflets, so replacement of the valve was performed. A biological mitral valve was used reinforced with teflon pledgetted sutures. The exposure and replacement of the valve was particularly difficult due to the small left atrial size. After completion of the replacement and during the atriotomy closure it was realized that the atriotomy was extended due to the retraction for better exposure of the valve, with subsequent injury to a) the RIPV, b) interatrial septum and c) posterior wall of the left atrium. The injured structures were repaired by prolene running sutures using teflon felts intermittently. After aortic clamp removal and during rewarming phase, a source of bleeding was noticed from the posterior wall of the right inferior pulmonary vein. Despite efforts to control the bleeding and repair the vein by adding interrupted sutures, the rupture was extended distally towards to the hilum of the right lung and also the vein's outlet to the atrium was significantly stenosed. The pump suction inserted in the left atrium and the full extension of the right inferior pulmonary vein rupture was then clearly exposed [Figure 1].

**Figure 2:**
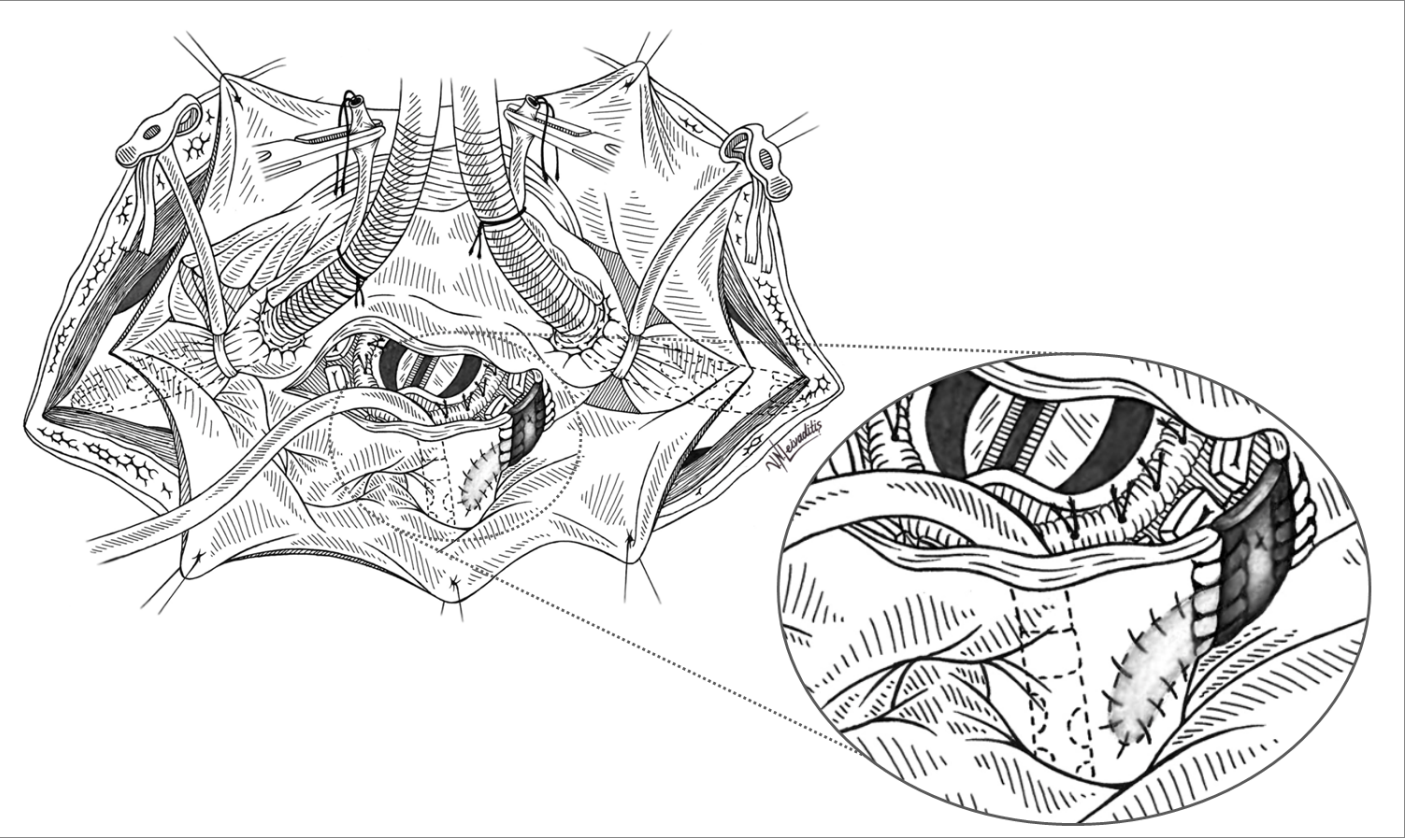
**Suture of the pericardial patch restores the rupture**.

**Figure 3:**
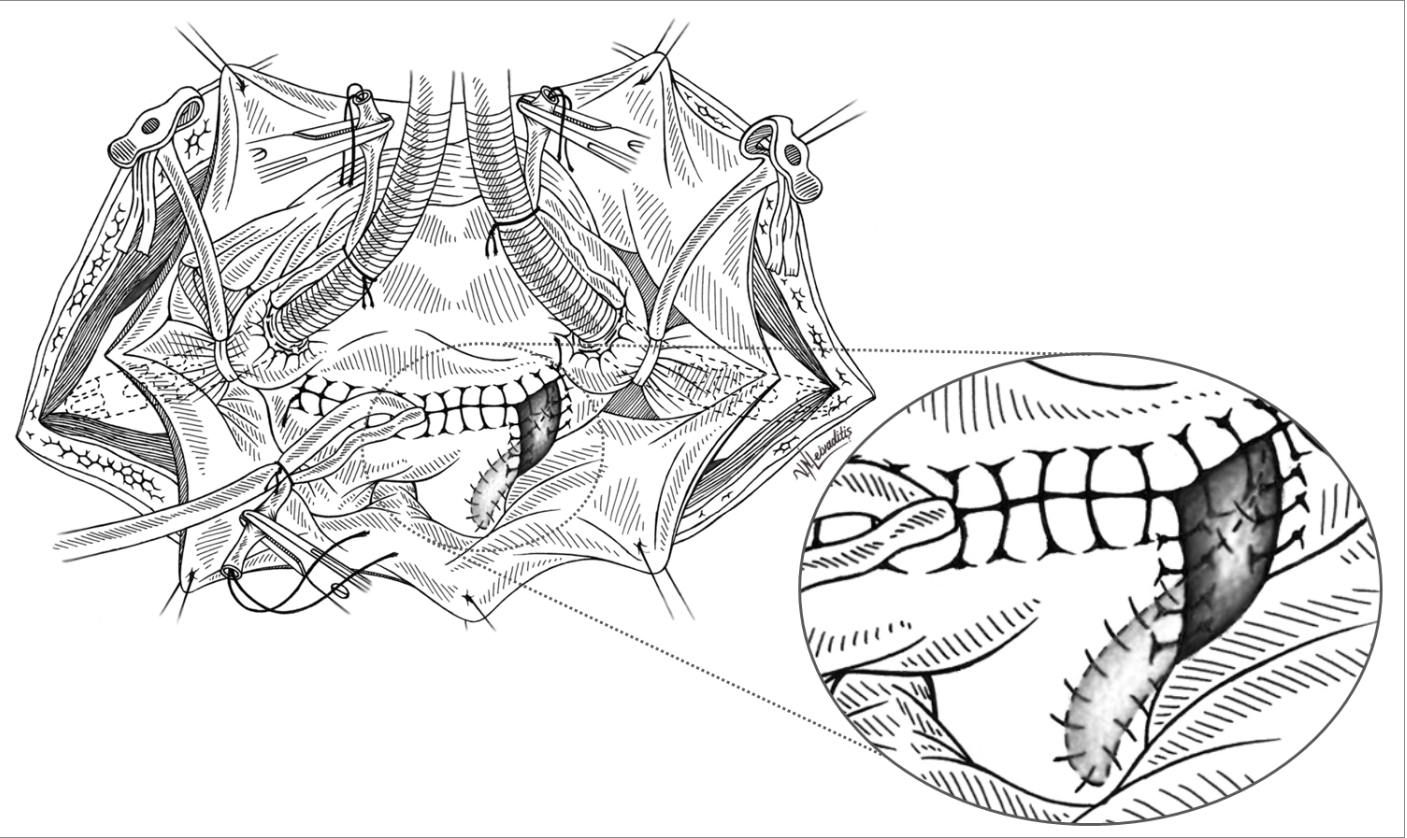
**Left atriotomy closure using continuous 4.0 prolene suture**. Before completing he suture the vent is reforwarded in the left ventricle via the prosthetic valve in order to perform the final deairing.

**Figure 4:**
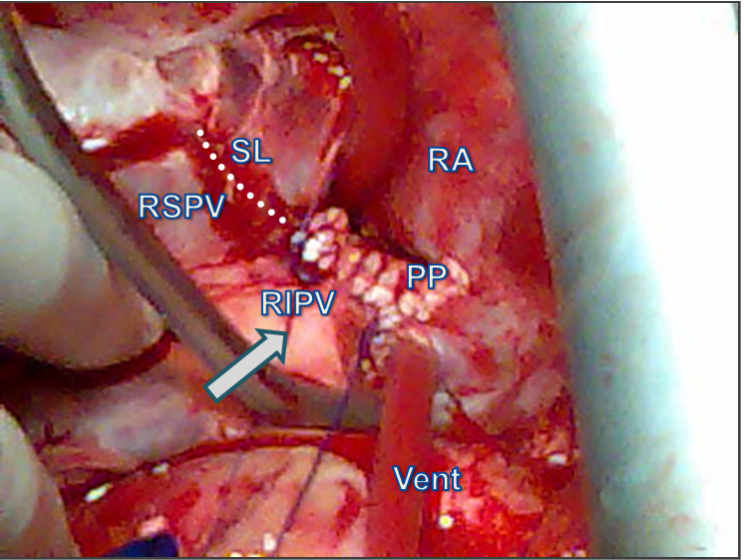
**Surgical field image showing the completed suture of the pericardial patch to the injured right inferior pulmonary vein.** RIPV: Right Inferior Pulmonary Vein (arrow), RSPV: Right Superior Pulmonary Vein, SL: Suture Line, PP: Pericardial Patch, RA: Right Atrium.

A small autologous pericardial patch (2 × 3 cm) was then excised and used for the repair starting from the distal posterior part of the rupture close to the right lung's hilum and ending in the proximal part of the vein's outlet in the left atrium [Figure 2]. The left atriotomy was completed by using continuous suture with 4-0 prolene, incorporating the trimmed end of pericardial patch [Figure 3, 4]. The result was satisfactory, the patient was disconnected from CPB and he had an uneventful postoperative course. He was extubated 16 hours later; he left ICU 24 hours postoperatively and was discharged from the hospital 6 days later. His chest X-Ray on discharge day was normal.

## Discussion

Injury of right inferior pulmonary vein or generally of right pulmonary veins during mitral valve surgery is reported extremely rare. In English literature, from 1970 to 2009, there are no references describing this rare complication. It should be possibly due to the fact that the cases of this injury are included in the general report as "postoperative bleeding" or "postoperative respiratory insufficiency". Indeed, the effort to suture the site of bleeding may cause an obstruction of right pulmonary vein, right pulmonary infarction, pulmonary edema, hypoxemia, and respiratory insufficiency [2]. Khonsari S and Sintek CF in their book noted that "because the atrial wall may be somewhat friable, excessive pull on the retractor may produce a sharing tear on the atrial wall edges, thus complicating closure [3]. It may be the sole possible reference in the literature about the referred complication but without any detailed suggestion about the repair or this complication.

Of note, a postoperative stenosis or obstruction of a pulmonary vein does not produce obvious clinical findings. Accidental total thrombosis of both pulmonary veins after ablation for AF may be misdiagnosed for 8-16 weeks [2,4]. The main symptoms of this complication such as shortness of breath, excertional dyspnea, and hypoxemia are usually attributed to the antecedent heart operation and/or to the pre-existent congestive heart failure. On the other hand, uncontrolled bleeding after discontinuation of by-pass increases the early operative mortality. The reasons of high mortality related to right pulmonary veins injuries are, in our opinion, the following: **a) **their wall is fine and friable and therefore suturing could be unsafe and further traumatic, **b) **the inevitable further sternal dilation in combination with pericardial suspension may increase the tension on the pulmonary veins and thus increases the friability during suturing, **c) **the intrapericardial length of the vein is very short and in combination with the proximity of inferior vena cava's outlet (witch entails the vena cannula) eliminates the space for manipulations and increases the difficulty of the repair, **d) **the proximity of site of bleeding to the right hilum may cause injuries to the other anatomic structures of the right lung during repair, **e) **The "visual contact" to the posterior surface of the RIPV is impossible especially after the atriotomy closure.

In our opinion, in order to prevent this possible complication, especially in patients with small left atrium, the integrity of the right pulmonary veins should be inspected just before completing the left atriotomy closure. In the case of an injured vein, instead of "blind suturing" of the bleeding's suture-line, a re-opening of the atriotomy-line should be performed, and it should be repaired by using a pericardial or even synthetic material graft. Autologous pericardial patch for easier suturing is the preferred material which should be incorporated in the completion of atriotomy closure. With this technique the tension to the vein's fragile wall is less and local stenosis can be avoided. The complication described can also be avoided by choosing other ways to access the mitral valve, such as via the right atrium (transeptal approach) or via the left atrium roof. These are some alternative options to the surgeon in cases of small left atrium or in cases where the anatomy of the pulmonary veins does not allow the surgeon to proceed through the classical Sondergaard's groove.

## Conclusion

To sum up, several useful conclusions can rise from this study and can be recapitulated in the following main points.

1) Mitral valve approach through the classical Sondergaard's groove must be chosen by the surgeon right after a short but close and careful view of the area's anatomy, focusing on the anatomy of the right pulmonary veins. This should also be a suitable option in cases of a large left atrium (>45 mm).

2) Left atriotomy needs to be tightly restored in order to avoid any possible postoperative leakage. However the atriotomy closure should always be performed respecting the permeability of the adjacent pulmonary veins' ostia.

3) Any hemorrhage of the anastomosis during atriotomy closure demands the precise location of it. High respect to the anatomic features of the region should always be a priority to the surgeon while restoring the bleeding area.

## Competing interests

The authors declare that they have no competing interests.

## Authors' contributions

EA conceived the idea, was the surgeon who performed the operation, wrote the first draft and led the project from beginning to end. VL assisted the study in data collection, literature review, draft revision, figure design and coordinating with all co-authors. AK helped with discussions about the topic and assistance in manuscript writing. PD provided expert opinion on this issue and also operated on this case. All authors critically read, discussed and approved the final draft of the manuscript.

## Consent

Written informed consent was obtained from the patient for publication of this case report and accompanying images. A copy of the written consent is available for review by the Editor-in-Chief of this journal.

## References

[B1] Byrne J Phillips B Cohn L Cohn L and Edmunds H Reoperative Valve Surgery Cardiac Surgery in the Adult 2003 2 MacGraw Hill Medical Publishing Division 1047 1054

[B2] Nehra D Liberman M Vagefi P Complete pulmonary venous occlusion after radiofrequency ablation for atrial fibrillation Ann Thorac Surg 2009 87 292 295 10.1016/j.athoracsur.2008.06.060 19101316

[B3] Khonsari S Sintek CF Cardiac Surgery safeguards and pitfalls in operative technique 2003 3 Philadelphia: Lippincott Williams & Wilkins

[B4] Qureshi A Prieto L Latson L Transcatheter angioplasty for acquired pulmonary vein stenosis after radiofrequency ablation Circulation 2003 108 1336 1342 10.1161/01.CIR.0000086322.21781.6A 12952852

